# Income inequality and alcohol attributable harm in Australia

**DOI:** 10.1186/1471-2458-9-70

**Published:** 2009-02-25

**Authors:** Paul M Dietze, Damien J Jolley, Tanya N Chikritzhs, Susan Clemens, Paul Catalano, Tim Stockwell

**Affiliations:** 1 Monash Institute of Health Services Research, Monash University, Clayton, Victoria, 3800, Australia; 2 Macfarlane Burnet Institute for Medical Research and Public Health (Burnet Institute), Melbourne, Victoria, 3004, Australia; 3 National Drug Research Institute, Perth, Western Australia, 6845, Australia; 4 Murdoch Children's Research Institute, Parkville, Victoria, 3052, Australia; 5 Centre for Addictions Research of British Columbia, University of Victoria, Victoria, British Columbia, Canada

## Abstract

**Background:**

There is little research on the relationship between key socioeconomic variables and alcohol related harms in Australia. The aim of this research was to examine the relationship between income inequality and the rates of alcohol-attributable hospitalisation and death at a local-area level in Australia.

**Method:**

We conducted a cross sectional ecological analysis at a Local Government Area (LGA) level of associations between data on alcohol caused harms and income inequality data after adjusting for socioeconomic disadvantage and remoteness of LGAs.

The main outcome measures used were matched rate ratios for four measures of alcohol caused harm; acute (primarily related to the short term consequences of drinking) and chronic (primarily related to the long term consequences of drinking) alcohol-attributable hospitalisation and acute and chronic alcohol-attributable death. Matching was undertaken using control conditions (non-alcohol-attributable) at an LGA level.

**Results:**

A total of 885 alcohol-attributable deaths and 19467 alcohol-attributable hospitalisations across all LGAs were available for analysis. After weighting by the total number of cases in each LGA, the matched rate ratios of acute and chronic alcohol-attributable hospitalisation and chronic alcohol-attributable death were associated with the squared centred Gini coefficients of LGAs. This relationship was evident after adjusting for socioeconomic disadvantage and remoteness of LGAs. For both measures of hospitalisation the relationship was curvilinear; increases in income inequality were initially associated with declining rates of hospitalisation followed by large increases as the Gini coefficient increased beyond 0.15. The pattern for chronic alcohol-attributable death was similar, but without the initial decrease. There was no association between income inequality and acute alcohol-attributable death, probably due to the relatively small number of these types of death.

**Conclusion:**

We found a curvilinear relationship between income inequality and the rates of some types of alcohol-attributable hospitalisation and death at a local area level in Australia. While alcohol-attributable harms generally increased with increasing income inequality, alcohol-attributable hospitalisations actually showed the reverse relationship at low levels of income inequality. The curvilinear patterns we observed are inconsistent with monotonic trends found in previous research making our findings incompatible with previous explanations of the relationship between income inequality and health related harms.

## Background

The harms caused by drinking are mediated by a variety of social and contextual factors operating at both individual and community levels. For example, social class (as represented by occupational categories) has been shown to independently account for the occurrence of alcohol caused harms [1]. Other socio-economic status variables such as income and spending power have also been shown to be associated with different types of alcohol caused death with lowered socioeconomic status often associated with higher likelihood of alcohol caused death [2]. Previous Australian work has demonstrated the extent to which these effects are evident in studies using data available at an ecologic level [3,4]. This work has shown that the relationship between drinking and alcohol caused hospitalisation in local areas is mediated by factors such as income levels and unemployment [3]. These findings in the alcohol literature are consistent with those evident in the wider field of social epidemiology where social contextual factors such as employment status, level of educational attainment an income have been shown to be related to a variety of health outcomes, such as mortality [5,6]. Again, lowered socio-economic status has generally been associated with poorer health outcomes, but this pattern does vary across specific conditions [7].

A considerable amount of work in social epidemiology has focused upon income inequality or disparity. Income disparities arise when income is unequally distributed across a given population, irrespective of the absolute income levels of the population. Income inequality has been shown to be associated with a variety of health and social outcomes including rates of all-cause mortality [8], violent crime [9], and life expectancy [see [10], for a review]. In general the pattern found is such that increases in income inequality are associated with increases in morbidity or mortality. However, the relationship between income inequality and health remains controversial, with some recent reviews and studies suggesting that the evidence of an association between income inequality and mortality is equivocal at best [10,11]. Nevertheless, some research by particular groups has continued to show an association between income inequality and measures of health [e.g. [12]].

Income inequality can be measured in a number of ways, but is most often measured through the Gini coefficient in the field of health research [13]. The Gini coefficient ranges from 0 (equitable income distribution) to 1 (maximum income inequality), representing the proportion of the population in specific income categories relative to the total population's income, and can be applied across time and place.

Various mechanisms have been postulated through which income inequality may manifest an effect on health outcomes [10,14]. Typically the postulated mechanisms are indirect in that income inequality is thought to be associated with social-contextual processes that may result in biased policy producing 'social capital' favouring the wealthy in an area. Such social capital may be expressed in policy terms such as better resource allocation, but may also reflect better social connectedness (e.g. having others who can be trusted) for those at higher income levels. Direct effects have also been postulated, whereby living near rich neighbours produces a kind of 'economic envy' among poorer people that results in greater stress and therefore poorer health (and possibly more drinking) [10]. These effects may be manifested in risk behaviours for poorer health such as alcohol or other drug consumption. These questions are of fundamental interest to public policy with recent debate in Australia, for example, focusing on questions of mechanisms for social improvement. In this debate understanding the interrelationships between social-contextual variables and health outcomes is seen as crucial [15]. Indeed, the recently-elected Australian Government has convened a National Preventive Health Taskforce that has a specific mandate in the area of alcohol harm reduction that sits within the Government's agenda on social inclusion [16].

Galea and colleagues have explored the relationship between income inequality and alcohol and other drug use and related harms (primarily) in New York City. Their work has shown positive associations between neighbourhood income inequality and drug overdose mortality [17], and alcohol and cannabis use [18], that were independent of other neighbourhood characteristics and individual-level variables such as personal income. However, as is the case with the general mortality studies reviewed by Lynch [11], there have been some inconsistent results in terms of the association between alcohol and drug related harms and income inequality. For example, Blomgren *et al *[19] found no significant association between area-level income inequality and alcohol caused mortality in a Finnish study. This apparent inconsistency between studies may be due to the small variability in income inequality evident in Finland (Gini coefficients ranging between 0.20 and 0.24). Indeed, the small variation in intra-country income inequalities in studied countries other than the USA has been proposed as an explanation for the mixed pattern of results found for income inequality in social epidemiology more broadly [10].

To our knowledge there has been no study of the relationship between income inequality and alcohol and drug related outcomes in Australia, despite the availability of a variety of alcohol and drug related data amenable to such an analysis. This research aimed to begin to address this gap by examining the relationship between income inequality and the rates of alcohol-attributable death and hospitalisation in Australia. On the basis of Galea *et al*'*s *findings we expected this relationship to be linear with increasing income inequality associated with increasing alcohol-attributable harm. As indicated, this relationship is important in the context of recent developments in Australia where understanding the relationship between social-contextual variables and health outcomes is firmly on the policy agenda.

## Methods

We used an ecologic design in which area-level variables were extracted from a series of datasets available from the Australian Bureau of Statistics (ABS) and the Australian Institute of Health and Welfare (AIHW). The research was approved by the Monash University Standing Committee on Ethics in Research involving Humans.

### Geographic units

We analysed data at the Local Government Area (LGA) level as LGA boundaries correspond to the administrative areas for which local governments are responsible. Local government is not only widely understood in defining community areas in Australia but also plays an important role in Australian alcohol policy; for example, in determining drinking by-laws, planning issues with respect to licensed premises, and safer city initiatives. This is also the level at which local community initiatives often operate [e.g. local liquor licensee accords [20]]. For these reasons the LGA was selected as the preferred geographic unit.

### Main outcome measures

Two types of alcohol related harm were examined; alcohol-attributable hospitalisation and alcohol-attributable mortality. Data on hospitalisations was obtained from the AIHW's National Hospital Morbidity Database (NHMD) which is a compilation of clinical information on hospital separations across all public and almost all private hospitals in Australia. Mortality data were sourced from the ABS Mortality Datafile, which is a compilation of details of all Australian deaths obtained from state and territory Death Registries. Both data sources contain information on age, sex, principal diagnosis, external cause and LGA of residence for all cases. Unfortunately the NHMD does not include detailed data on place of residence for Queensland or South Australian hospitalisations, meaning that these two states were not included in the analysis of alcohol-attributable hospitalisations. Principle diagnosis and any applicable external causes are coded on both datasets according to International Classification of Diseases *10*^*th*^*revision*, Australian Modification (ICD-10-AM). Hospitalisation data analysed in this report cover the 1999/2000 fiscal year while the mortality data were obtained for the 2000 calendar year.

Aggregate measures of hospitalisation and death attributable to risky/high-risk drinking at an LGA level were developed by first extracting cases with a principle or external cause diagnosis indicating the cause of ill-health or death was wholly attributable to alcohol. These conditions were taken from those identified in a meta-analysis originally published by English *et al *[21] These cases were then categorised as acute (primarily related to the effects of risky drinking in the short-term, e.g. alcoholic beverage poisoning) or chronic (primarily related to the effects of risky drinking in the long-term, eg alcoholic liver cirrhosis) on the basis of the likely drinking pattern that resulted in hospitalisation or death, as recommended by the World Health Organisation [22]. We also extracted a series of cases identified as largely unrelated to drinking. For hospitalisations, these were acute appendicitis, diverticulitis, hyperplasia of prostate, genital prolapse and osteoarthritis – each identified as non-alcohol-related by previous Australian research [3]. Causes of death which are known to be largely unaffected by alcohol consumption are relatively rare in Australia, for mortality data therefore, controls were defined as cases for which the alcohol aetiologic fraction for risky/high-risk drinking was zero but which may have attracted an alcohol aetiologic fraction for low-risk drinking (e.g. ischaemic heart disease).

Once extracted, these measures were aggregated by Australian LGAs. They were then age and sex standardised via indirect standardisation using estimated resident populations from the 2001 census. Initial inspection of the outcome data showed that the resultant standardised morbidity ratios (SMRs) were highly skewed at an LGA level for both alcohol-attributable and control conditions. As a consequence these data were log-transformed and then a matched rate ratio was generated for each measure (acute and chronic) of hospitalisation and death. This matched rate ratio was the log-transformed rate of the alcohol SMR divided by the control SMR for each LGA.

### Predictor variables

Three predictor variables were developed for LGAs and included as predictors. First, a Gini coefficient was developed using detailed reported weekly income information obtained from the 2001 ABS census. Second, the socioeconomic characteristics of areas were indexed through the ABS *Socio-Economic Index for Areas, SEIFA *disadvantage score [23]. This score is census-derived and summarises the socioeconomic disadvantage of areas focusing on the following area-level characteristics: low income earners; relatively lower educational attainment; and high unemployment. Low scores show high levels of disadvantage while high scores show relatively low levels of disadvantage within areas. Third, the geographic characteristics of areas were indexed through the ABS *Accessibility/Remoteness Index for Australia*, *ARIA *score [24]. This index summarised area-level characteristics for census collector districts (CDs) in terms of the distance of the CD from access to the widest range of goods and services and opportunities for social interaction. Mean ARIA scores can be calculated for LGAs (compilations of CDs) and then classified according to the following five categories developed by the ABS: Major cities; Inner Regional Australia; Outer Regional Australia; Remote Australia; and Very Remote Australia.

Gini coefficients are calculated as the area under a Lorenz curve plotted with income categories on the x-axis and the proportion of the total population's income on the y-axis). In this study we generated Gini coefficients for LGAs in the following way. First, the number of households within each LGA in each income category was determined by aggregating across the number of residents and the type of household (e.g. lone person, group, one family, two families). The midpoint of each income category for each LGA was then multiplied by the number of households in each income category in the LGA. However, the income data collected in the Australian census is right-censored because the largest response category available is $2000. In order to provide a more parsimonious estimate of the midpoint of this income category we chose a value of $2250, consistent with the midpoints of other income categories. However, the true midpoint of this category is still likely to be underestimated. This right-censoring means that the resultant income distribution will probably show less variation and produce more conservative estimates of the Gini coefficient than if higher income values were available. Income categories within LGA were then ranked to form progressive cumulative totals of numbers of households at each income level. We then numerically integrated the Lorenz curve of cumulative income vs cumulative households, using a simple trapezoidal rule algorithm. For each LGA, a Gini coefficient was then calculated as the difference between 0.5 and the computed area under the LGA's Lorenz curve.

**Table 1:** Descriptive statistics for the key variables included in analyses

**Variable**	**Total**	**LGA Mean**	**Min**	**Max**	**N LGAs**
Population	18726897	29725	85	880519	630
SEIFA disadvantage	na	982.3	406.4	1151.5	630
Gini coefficient	na	.181	.105	.280	580
ARIA score	na	4.145	0	15	624
Major cities		0.015	0	0.160	103(17%)
Inner regional		1.295	.201	2.389	145(23%)
Outer regional		3.962	2.424	5.912	230(37%)
Remote		7.667	5.966	10.469	77(12%)
Very remote		12.974	10.731	15	69(11%)
Morbidity					
Number acute wholly-alcohol-caused	9317	21.8	1	211	
Number chronic wholly- alcohol-caused	10150	23.7	1	281	
Number controls	64654	151.1	1	1304	
*(Gini coefficient used)*	na	.*183*	.*119*	.*280*	
Mortality					
Number acute wholly-alcohol- caused	170	0.3	1	5	
Number chronic wholly- alcohol-caused	715	1.3	1	16	
Number controls (acute)	136	0.2	1	7	
Number controls (chronic)	11161	19.9	1	191	
*(Gini coefficient used)*	na	.*182*	.*105*	.*280*	

### Data analysis

All analyses were conducted using Stata/SE V9. The matched rate ratios for all four alcohol-attributable outcomes (acute and chronic alcohol-attributable hospitalisations, acute and chronic alcohol-attributable deaths) described above were entered into a linear regression as outcome variables with LGA-level Gini coefficient, SEIFA disadvantage scores (included as decile values) and ARIA category (five levels) entered as predictor variables. The GINI coefficient was centred (i.e. X = Gini-mean(Gini)) in order to minimise the correlation between coefficients. There was a large variation evident in the number of cases occurring in LGAs, related in part to the size of the LGAs. In order to control for these variations in the models we weighted the models by the number of cases (both alcohol-attributable and control) using the analytic weights procedure available in Stata. This weighting involves using the generalised inverse variance for each LGA in order to account for variation in LGA size. Initial exploration suggested that the relationship between the centred Gini coefficient and all outcomes was curvilinear and so we included a quadratic term in the regression analyses. We also attempted to control for spatial autocorrelation. However, technical difficulties of adjusting for spatial correlation in a weighted analysis precluded any adjustment for these effects. Nevertheless, the effects of spatial autocorrelation are typically small or non-existent in previous studies of alcohol-attributable harm [e.g. [25]].

There was a total of 630 Australian LGAs included in our dataset. The number of events varied across LGAs with some LGAs having few or zero cases or controls. LGAs with zero cases or controls for a given outcome measure were not included in the analysis of that outcome. As hospitalisation data from Queensland and South Australia were unavailable, LGAs from these two states were not included in the analysis of alcohol-attributable hospitalisations. The number of LGAs included in each analysis is specified in relation to each of the models below.

### Results

There were 885 alcohol-attributable deaths and 19467 alcohol-attributable hospitalisations across all LGAs. Table 1 shows the major descriptive characteristics of the LGA-level data included in the analysis across all of the diagnostic categories, along with the Gini coefficients included in the different analyses undertaken. The mean value of the Gini coefficient (the area of the curve deviating from the diagonal) was around 0.18, ranging from .105 (most equitable) to 0.28 (most inequitable). The remainder of the results section describes the results of the regression analyses undertaken in relation to each specific outcome measure, as described above. A sensitivity analysis in which we excluded small and large LGAs by including only the middle two quartiles of LGAs by population size showed a more-or-less identical pattern to the results presented below, and is therefore not reported here 1.

### Alcohol-attributable hospitalisation

There was a relatively large number of alcohol-attributable hospitalisations and 373 LGAs with corresponding Gini coefficients available for analysis of the acute alcohol-attributable hospitalisations and 349 LGAs with corresponding Gini coefficients available for analysis of the chronic alcohol-attributable hospitalisations. Table 2 shows that there was a highly significant association between the squared centred Gini coefficient and the rate ratio of both acute and chronic hospitalisations at an LGA level, after adjusting for SEIFA disadvantage and ARIA scores. The ARIA scores also showed an interesting curvilinear pattern with decreased rates of acute and chronic alcohol-attributable hospitalisation for inner and outer regional areas in comparison to metropolitan areas and increased rates in the remote and very remote areas.

**Table 2:** Regression coefficients and 95% CIs for the predictor variables included in the model for alcohol-attributable hospitalisations

	Acute – overall *R*^2 ^= .*143* *(N LGAs = 374)*				Chronic- overall *R*^2 ^= .*112* *(N LGAs = 350)*			
	Beta	P	LCI	UCI	Beta	P	LCI	UCI

Gini	22.36	0.00	14.50	30.21	18.59	0.00	9.01	28.16
Centred Gini-squared	389.40	0.00	203.99	574.82	379.14	0.00	156.34	601.94
SEIFA disad decile 1	ref				ref			
SEIFA disad decile 2	-0.04	0.76	-0.33	0.24	0.28	0.10	-0.05	0.62
SEIFA disad decile 3	-0.10	0.52	-0.40	0.20	0.22	0.23	-0.14	0.58
SEIFA disad decile 4	-0.09	0.57	-0.38	0.21	0.00	1.00	-0.35	0.35
SEIFA disad decile 5	-0.06	0.68	-0.34	0.22	-0.03	0.86	-0.37	0.31
SEIFA disad decile 6	-0.20	0.14	-0.47	0.07	0.27	0.10	-0.05	0.59
SEIFA disad decile 7	-0.10	0.54	-0.44	0.23	0.51	0.01	0.12	0.90
SEIFA disad decile 8	-0.09	0.51	-0.38	0.19	0.16	0.35	-0.18	0.50
SEIFA disad decile 9	-0.28	0.04	-0.55	-0.02	0.33	0.04	0.01	0.64
SEIFA disad decile 10	0.09	0.53	-0.20	0.38	0.61	0.00	0.27	0.96
ARIA – major cities	ref				ref			
ARIA – inner regional	-0.21	0.00	-0.34	-0.08	-0.32	0.00	-0.48	-0.17
ARIA – outer regional	-0.11	0.26	-0.30	0.08	-0.27	0.02	-0.49	-0.05
ARIA – remote	0.49	0.08	-0.05	1.03	0.42	0.24	-0.29	1.12
ARIA – very remote	1.50	0.00	1.00	2.00	0.90	0.01	0.24	1.56
_cons	0.07	0.53	-0.14	0.28	-0.26	0.05	-0.51	0.00

**Figure 1:**
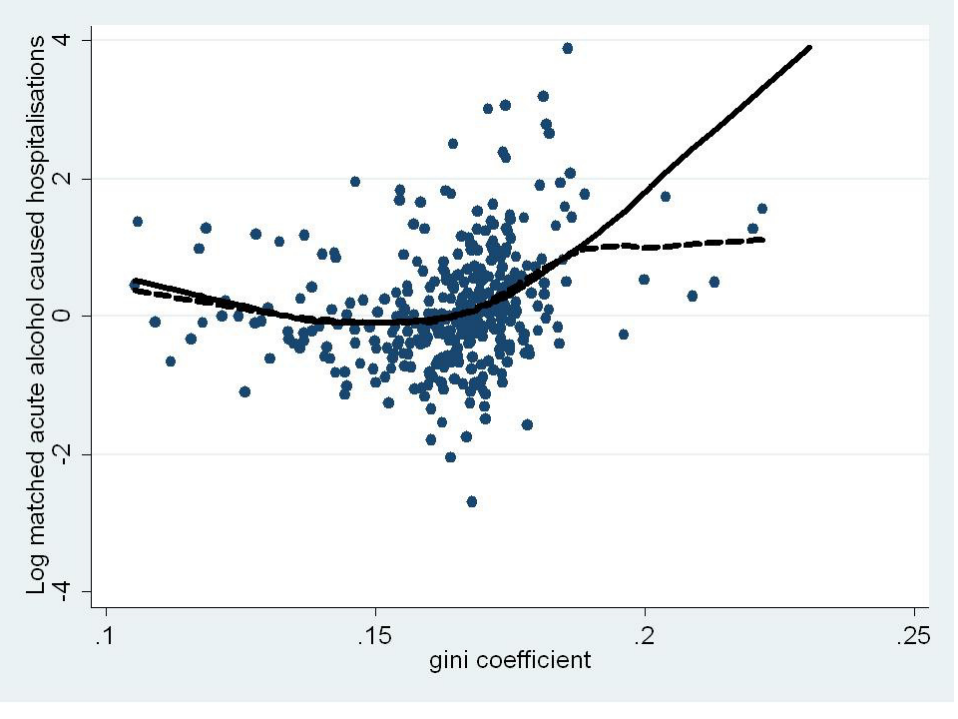
**Gini coefficient by matched rate ratio for acute alcohol-attributable hospitalisations for Australian LGAs in 99/00 fiscal year **(trendlines show Loess curves of best fit for model predicted, (solid) and raw scores (dashed)).

**Figure 2:**
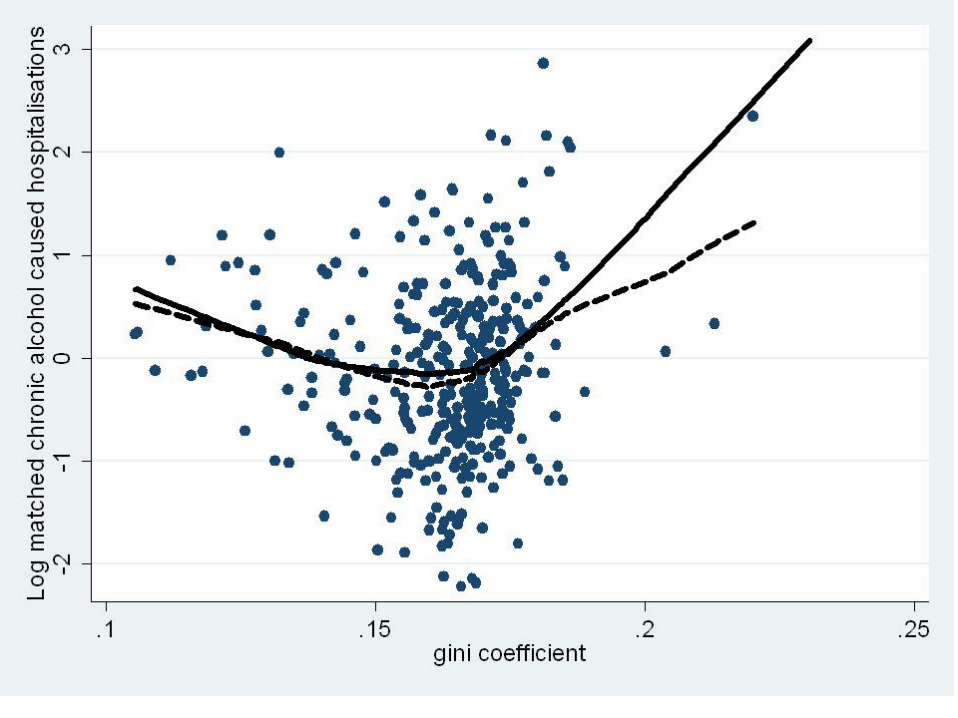
**Gini coefficient by matched rate ratio for chronic alcohol-attributable hospitalisations for Australian LGAs in 99/00 fiscal year **(trendlines show Loess curves of best fit for model predicted, (solid) and raw scores (dashed)).

Figures 1 and 2 show the raw scores as well as trend lines (plotted using Loess curves of best fit) of the model-predicted and raw scores for the acute (Figure 1) and chronic (Figure 2) alcohol-attributable hospitalisations. The curvilinear quadratic relationships were very similar such that with increasing inequality, the rate ratio first decreased but increased dramatically as the Gini coefficient approached 0.2 for both acute and chronic alcohol-attributable hospitalisations.

### Alcohol-attributable deaths

Table 1 shows that, compared to alcohol-attributable hospitalisations, there was a much smaller number of alcohol-attributable deaths available for analysis. However, while there were relatively few alcohol caused deaths, there was a larger number of LGAs with corresponding Gini coefficients available for analysis. Table 3 shows that there was a highly significant association between the squared centred Gini coefficient and the rate ratio of the chronic, but not the acute, alcohol-attributable deaths. Interestingly, the SEIFA disadvantage scores were associated with acute alcohol-attributable deaths with the most disadvantaged decile having higher rate ratios than the remaining deciles, significantly so in comparison to deciles 2–4 (areas of relatively high disadvantage). In comparison to the Major Cities, the ARIA scores for all other areas were associated with fewer chronic alcohol-attributable deaths.

**Table 3:** Regression coefficients and 95% CIs for the predictor variables included in the model for alcohol-attributable deaths

	Acute- overall *R*^2 ^= .*181* *(N LGAs = 499)*				Chronic- overall *R*^2 ^= .*153* *(N LGAs = 180)*			
	Beta	P	LCI	UCI	Beta	P	LCI	UCI

Gini	3.97	0.64	-12.60	20.54	30.70	0.00	21.66	39.73
Centred Gini-squared	113.42	0.73	-539.57	766.41	374.72	0.00	145.72	603.73
SEIFA disad decile 1	ref				ref			
SEIFA disad decile 2	-1.33	0.00	-2.04	-0.62	-0.42	0.02	-0.76	-0.07
SEIFA disad decile 3	-0.80	0.02	-1.50	-0.11	-0.13	0.44	-0.45	0.19
SEIFA disad decile 4	-0.93	0.01	-1.65	-0.22	-0.19	0.26	-0.53	0.14
SEIFA disad decile 5	-0.25	0.53	-1.01	0.52	0.06	0.74	-0.28	0.40
SEIFA disad decile 6	-0.70	0.04	-1.39	-0.02	-0.23	0.16	-0.54	0.09
SEIFA disad decile 7	-0.50	0.31	-1.47	0.47	0.08	0.69	-0.30	0.45
SEIFA disad decile 8	-0.21	0.64	-1.10	0.68	0.20	0.26	-0.15	0.55
SEIFA disad decile 9	-0.17	0.65	-0.93	0.59	-0.08	0.65	-0.41	0.26
SEIFA disad decile 10	-0.02	0.96	-0.74	0.70	-0.01	0.93	-0.36	0.33
ARIA – major cities	ref				ref			
ARIA – inner regional	-0.54	0.01	-0.92	-0.15	-0.27	0.00	-0.43	-0.11
ARIA – outer regional	-0.06	0.82	-0.57	0.45	-0.36	0.00	-0.56	-0.16
ARIA – remote	0.38	0.41	-0.52	1.27	-0.67	0.01	-1.16	-0.18
ARIA – very remote	0.13	0.73	-0.61	0.86	-0.57	0.08	-1.21	0.06
_cons	0.55	0.06	-0.03	1.14	0.11	0.42	-0.16	0.38

**Figure 3:**
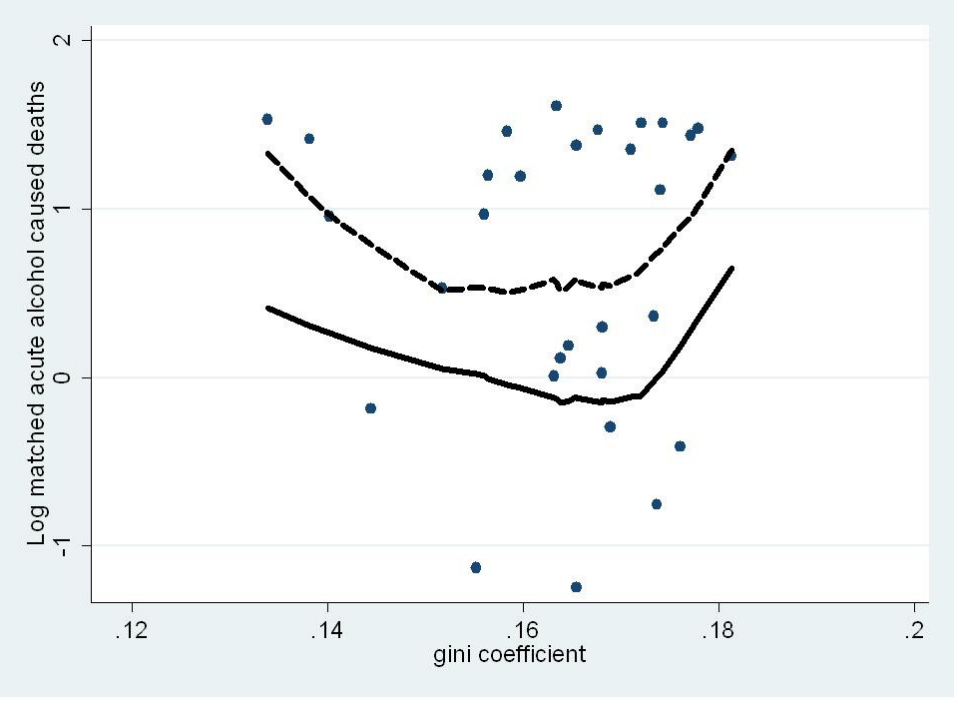
**Gini coefficient by matched rate ratio for acute alcohol-attributable deaths for Australian LGAs in 0001 fiscal year **(trendlines show Loess curves of best fit for model predicted, (solid) and raw scores (dashed)).

**Figure 4:**
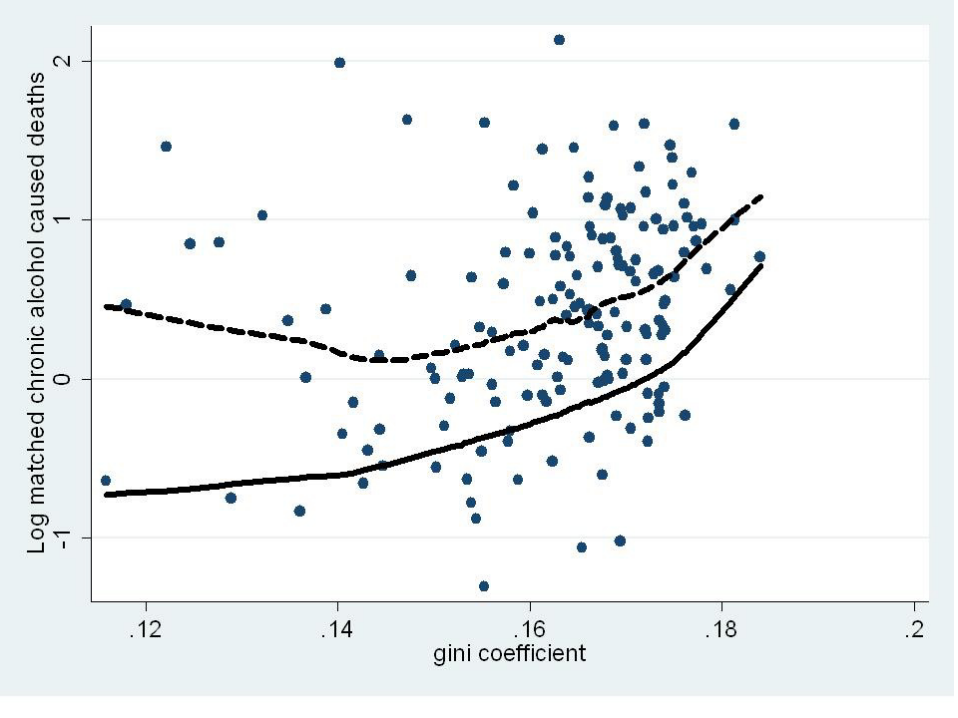
**Gini coefficient by matched rate ratio for chronic alcohol-attributable deaths for Australian LGAs in 00/01 fiscal year (trendlines show Loess curves of best fit for model predicted, (solid) and raw scores (dashed))**.

Figures 3 and 4 show the raw scores as well as trend lines (plotted using Loess curves of best fit) of the model-predicted and raw scores for the acute (Figure 1) and chronic (Figure 2) alcohol caused deaths. Figure 3 highlights not only the fact that no clear relationship was evident between the Gini coefficient and acute alcohol-attributable death but also just how sparse the data were in comparison to the other measures of alcohol-attributable harm. In contrast, Figure 4 shows the curvilinear quadratic relationship between the Gini coefficient and chronic alcohol-attributable death. However, unlike the hospitalisation data shown in Figures 1 and 2, there was no evidence of the concave decrease, with a flat relationship evident until values of the Gini coefficient reach about .17, above which the increase appears to follow a similar pattern to the hospitalisation data shown in Figures 1 and 2.

## Discussion

This study is the first to provide evidence of a relationship between income inequality and alcohol-attributable harm in Australia. The nature of the relationship was consistent across acute and chronic alcohol-attributable hospitalisations and was similar for chronic alcohol-attributable deaths. In general the results showed that increasing LGA-level income inequality was associated with increasing rates of alcohol-attributable harm, after adjusting for general socio-economic disadvantage and remoteness of LGAs. While these relationships appeared strong and robust, there was no evidence of a relationship between income inequality and acute alcohol-attributable deaths, possibly due to the relatively small number reported.

The relationship between income inequality and health outcomes such as all-cause mortality remains controversial [10,14]. However, observed relationships have typically been shown to be a monotonically increasing function; that is, as income inequality increases so too do rates of ill-health or death [13]. In this context our findings of a curvilinear function were unexpected; especially the apparent decline in rates of alcohol-attributable harm with initial increases in income inequality. In contrast, the significant relationship between income inequality and chronic alcohol-attributable death appeared to follow a pattern similar to that found by Galea *et al *[17] in relation drug overdose. However, Galea *et al's *matched analysis (where they included other injury death as controls) showed a clear monotonic trend with no statistically significant differences in odds between different percentiles of their Gini coefficient. In this way even our findings in relation to the association between the Gini coefficient and mortality differ from those found in previous research.

As indicated, the relationship between income inequality and poor health has been potulated to result from a variety of direct or indirect causal paths [10,14]. Processes such as 'economic envy' may explain the area-level increases in adjusted rates of alcohol-attributable harms observed in our study at the upper-end of our Gini coefficient. However, neither direct nor indirect pathways can explain the observed decline in the rate of alcohol-attributable hospitalisation at the lower values of the Gini coefficient. It is unlikely that 'economic envy' of neighbours would be worse for those areas of lesser inequality and it is also unlikely that other forms of social capital would be lower in these areas, unless there are some unknown confounding factors for which we did not control. One candidate explanation may be the rapid development of the urban fringe around Australia's cities which are typically homogenous with respect to a variety of socio-economic characteristics. However, many of these areas would be classified as Inner-Regional on the ARIA index and our findings with respect to income inequality were robust after adjusting for variations in remoteness. An alternative may be that the homogeneity in areas with low levels of inequality may produce communities with low levels of diversity and that some smaller increments in income inequality may produce communities with more diversity and therefore more interest for community members. The impact of this diversity on drinking behaviours and health outcomes requires further examination using richer data than that available for this study. Irrespective, it is difficult to formulate direct policy recommendations (e.g. interventions designed to reduce income inequalities) on the basis of our findings as direct intervention to affect income inequality may indeed increase the rate of alcohol-attributable harms – at least at lower levels of inequality.

The findings in relation to remoteness showed that inner and outer-regional areas of the country were less likely to experience alcohol-attributable harm than major cities and remote areas of Australia. These geographic variations contrast with previous Australian research that has generally shown metropolitan areas to have lower rates of alcohol-attributable harm than non-metropolitan areas [26]. This contrast may derive from our inclusion of additional levels of remoteness within the non-metropolitan classification. This suggests that the geographic variation observed in these previous Australian studies was probably driven largely by the remote and very remote areas of the country included in the 'non-metropolitan' categories used. Our finer specification has important implications, suggesting the need for targeting alcohol programs and policy towards remote and very remote areas of the country.

This study has several limitations. First, the study was ecological meaning that there is the potential for ecological bias. However, the Gini coefficient, our exposure variable, is not subject to a classical ecological fallacy because it is a characteristic the LGA that has been measured. Second, the study was cross-sectional in nature. Ready interpretation of cross-sectional ecological studies requires the exposure (e.g. drinking, income inequality) and outcome (e.g. hospitalisation, death) to occur within a similar timeframe. In this framework it is reasonable to infer that hazardous/high-risk drinking produced the acute alcohol-attributable outcome for which a relationship to income inequality was observed (for hospitalisation at least). The same is not necessarily true of the chronic alcohol-attributable conditions that result from sustained patterns of risky/high-risk drinking over time. In this case we need to assume relative stability in persons' residence over time, and relatively static levels of inequality. The validity of these assumptions is unknown.

## Conclusion

In this study we found a curvilinear relationship between income inequality and the rates of some types of alcohol-attributable hospitalisation and death at a local area level in Australia. While alcohol-attributable harms generally increased with increasing income inequality, alcohol-attributable hospitalisations actually showed the reverse relationship at low levels of income inequality. These curvilinear patterns we observed are inconsistent with monotonic trends found in previous research making our findings incompatible with previous explanations of the relationship between income inequality and health related harms. This has significant implications for public policy initiatives directed towards reducing income inequalities within LGAs, as it suggests that any effects on alcohol-attributable harms may not be uniform across different levels of income inequality.

## Competing interests

The authors declare that they have no competing interests.

## Authors' contributions

All authors contributed to the design of the study. PD led the writing of the manuscript and DJ and PD led the statistical analyses. TC and PC extracted the data for analysis. All authors contributed to the drafting of the manuscript. All authors read and approved the final manuscript.

## Pre-publication history

The pre-publication history for this paper can be accessed here:


